# Hyperbaric oxygen in the artificial intelligence era: integration and innovation

**DOI:** 10.4103/mgr.MEDGASRES-D-25-00234

**Published:** 2026-05-21

**Authors:** Francisco Epelde

**Affiliations:** Internal Medicine Department, Parc Taulí Hospital Universitari, Institut d’Investigació i Innovació Parc Taulí (I3PT-CERCA), Universitat Autònoma de Barcelona, Sabadell, Spain

**Keywords:** artificial intelligence, biosensors, digital twin, hyperbaric oxygen therapy, machine learning, precision medicine

## Abstract

Hyperbaric oxygen therapy is established for decompression illness, carbon monoxide poisoning, radiation-induced tissue injury, and diabetic foot ulcers. Interest in neurological and inflammatory indications is growing, yet outcomes are constrained by heterogeneous protocols, limited patient selection tools, and the absence of real-time physiological guidance. Artificial intelligence, including machine learning, explainable artificial intelligence, and digital twins, has transformed other clinical domains and could enable precision hyperbaric oxygen therapy. We searched PubMed, Scopus, Web of Science, and Google Scholar (January 2000–March 2025). Terms included “hyperbaric oxygen therapy” and (“artificial intelligence” OR “machine learning” OR “deep learning” OR “digital twin” OR “biosensor*”). English-language, peer-reviewed clinical, preclinical, or computational studies explicitly linking hyperbaric oxygen therapy with artificial intelligence or enabling technologies were eligible. Editorials without primary data, abstracts without full text, and non-peer-reviewed sources were excluded. A Preferred Reporting Items for Systematic Reviews and Meta-Analyses-style flow-guided screening and selection. Fifty-three eligible studies (≥30% from 2022–2025) illustrate four convergent application areas: (1) protocol optimization using multicenter data, (2) biomarker-driven patient selection via multi-omics and imaging, (3) real-time adaptive control using biosensors, and (4) predictive safety analytics for oxygen toxicity and barotrauma. Exemplars from radiology, critical care, and cardiology demonstrate the feasibility of decision support, federated learning, and digital-twin simulations adaptable to hyperbaric oxygen therapy. Integrating artificial intelligence with hyperbaric oxygen therapy can shift practice from empirical protocols to patient-tailored, data-informed therapy. Priorities include hybrid clinical trials, interoperable registries, explainable and bias-aware models, and regulatory-aligned validation. With rigorous governance, artificial intelligence can improve efficacy, safety, and efficiency while advancing mechanistic understanding of hyperbaric oxygen therapy.

## Introduction

Hyperbaric oxygen therapy (HBOT) administers 100% oxygen at elevated pressures, commonly 1.3 to 3.0 atmospheres absolute, thereby increasing the partial pressure of dissolved oxygen in plasma and steepening diffusion gradients across hypoxic tissue. Clinically, HBOT is a mainstay for decompression illness, arterial gas embolism, and carbon monoxide poisoning, and serves as an adjunct for difficult-to-heal wounds and late radiation tissue injury.^1^,^2^,^3^,^4^,^5^ All HBOT dose bands are hypothesis-generating and not prescriptive; these are summarized in **Tables 1–4**.

**Table 1:** Evidence-to-decision summary for HBOT in PTSD

Criterion	Summary
Study base (design/size)	Small heterogeneous RCTs and observational cohorts; variable protocols and timing; limited sham-controlled data.
Main comparators	Standard care; psychotherapy/pharmacotherapy; occasional sham HBOT in select RCTs.
Outcomes signal	Mixed but suggestive improvements in symptom scores (e.g., CAPS-like measures) in some studies; durability uncertain.
Risk of bias (overall)	Moderate to high (heterogeneity, small samples, and potential unblinding).
Certainty of evidence (GRADE-like)	Low.
Harms/safety considerations	Barotrauma risk low-moderate; central nervous system oxygen toxicity rare with monitoring; psychological tolerability generally acceptable.
Resource use/feasibility	Requires chamber access and trained staff; schedule adherence can be challenging in PTSD populations.
Proposed dose bands status	Hypothesis-generating pending prospective validation.

CAPS: Clinician-Administered PTSD Scale; GRADE: Grading of Recommendations Assessment, Development, and Evaluation; HBOT: hyperbaric oxygen therapy; PTSD: posttraumatic stress disorder; RCT: randomized controlled trial.

**Table 2:** Evidence-to-decision summary for HBOT in late radiation tissue injury

Criterion	Summary
Study base (design/size)	Multiple RCTs and controlled series for ORN and pelvic radiation injury; moderate cumulative sample sizes.
Main comparators	Standard care (surgery, dental care, and wound care); sometimes sham or alternative dose regimens.
Outcomes signal	Clinical benefit signals in pain, wound healing, and tissue viability in several trials; effect size varies by site.
Risk of bias (overall)	Low to moderate.
Certainty of evidence (GRADE-like)	Moderate.
Harms/safety considerations	Ear/sinus barotrauma the most common; careful pre-dental/surgical planning mitigates risks.
Resource use/feasibility	Feasible in experienced centers; cost offset possible if surgical complications decline.
Proposed dose bands status	Hypothesis-generating pending prospective validation.

GRADE: Grading of Recommendations Assessment, Development, and Evaluation; HBOT: hyperbaric oxygen therapy; ORN: osteoradionecrosis; RCT: randomized controlled trial.

**Table 3:** Evidence-to-decision summary for HBOT in diabetic foot ulcers

Criterion	Summary
Study base (design/size)	Mixture of RCTs, cohorts, and registry analyses; protocol heterogeneity; wound-care cointerventions common.
Main comparators	Advanced wound care, revascularization where indicated, offloading; occasional sham HBOT.
Outcomes signal	Some studies show improved healing rates and limb salvage; others neutral; effect may depend on ischemia/infection phenotype.
Risk of bias (overall)	Moderate (cointerventions, selection).
Certainty of evidence (GRADE-like)	Low to moderate.
Harms/safety considerations	Similar to general HBOT; glycemic control and wound infection monitoring are key.
Resource use/feasibility	High session counts; logistical burden but potentially cost-effective in selected high-risk ulcers.
Proposed dose bands status	Hypothesis-generating pending prospective validation.

GRADE: Grading of Recommendations Assessment, Development, and Evaluation; HBOT: hyperbaric oxygen therapy; RCT: randomized controlled trial.

**Table 4:** Evidence-to-decision summary for HBOT in necrotizing soft tissue infection

Criterion	Summary
Study base (design/size)	Predominantly retrospective cohorts and case series; no large definitive RCTs.
Main comparators	Standard aggressive surgical debridement + antibiotics + intensive care; without HBOT.
Outcomes signal	Associations with reduced mortality/complications suggested in some cohorts; confounding likely.
Risk of bias (overall)	High (confounding by indication, center experience).
Certainty of evidence (GRADE-like)	Very low to low.
Harms/safety considerations	Transport to chamber and hemodynamic stability must be ensured; close monitoring for oxygen toxicity.
Resource use/feasibility	Feasibility depends on proximity to chamber and ICU coordination; time-sensitive logistics.
Proposed dose bands status	Hypothesis-generating pending prospective validation.

GRADE: Grading of Recommendations Assessment, Development, and Evaluation; HBOT: hyperbaric oxygen therapy; ICU: intensive care unit; RCT: randomized controlled trial.

Over the past decade, research has probed potential benefits in neurological and inflammatory diseases, including post-concussive syndromes, stroke recovery, and inflammatory bowel disease.^6^,^7^,^8^ Across these domains, HBOT is thought to modulate inflammatory signaling, improve microvascular dynamics, and, in the central nervous system, and support neuroplastic remodeling.

Despite these encouraging signals, the practice remains largely empirical. Protocols differ across centers in pressure, session duration, and the number of exposures, complicating meta-analysis and guideline harmonization.^5^,^9^ Real-time monitoring inside chambers is limited; most programs rely on routine vital signs rather than continuous physiologic feedback. Patient selection also leans on clinical heuristics (e.g., wound severity, prior radiation dose) rather than validated biomarkers.

In parallel, artificial intelligence (AI) has become a central force in clinical medicine. From image interpretation to prognostic modeling and treatment optimization, AI demonstrates the capacity to extract patterns from high-dimensional data and to generate actionable predictions.^10^,^11^,^12^,^13^ Explainable AI methods enable transparency, while digital twins — patient-specific computational replicas — are emerging as tools to simulate clinical scenarios ahead of intervention.^14^,^15^,^16^ This review argues that AI can tackle core limitations of HBOT: protocol heterogeneity, unpredictable response, limited safety prediction, and absent in-chamber adaptation. Classic emergencies — decompression illness, arterial gas embolism, and carbon monoxide poisoning — are acknowledged. Our deliberate focus is on longer-course indications where AI-assisted dose governance, session-level monitoring, and learning registries are most impactful.

## Search Methodology

A structured literature search was conducted in PubMed, Scopus, Web of Science, and Google Scholar (January 2000–March 2025). We combined “hyperbaric oxygen therapy” (HBOT) with AI-related terms (“artificial intelligence,” “machine learning,” “deep learning,” “digital twin,” “biosensor*”). We included peer-reviewed English-language clinical, preclinical, or computational studies explicitly linking HBOT to AI or enabling technologies (e.g., wearable sensors used to inform algorithms). We excluded abstracts without full text, non-peer-reviewed sources, and editorials without data. Preferred Reporting Items for Systematic Reviews and Meta-Analyses methodology was used. Results are presented in **Figure 1**.

**Figure 1:**
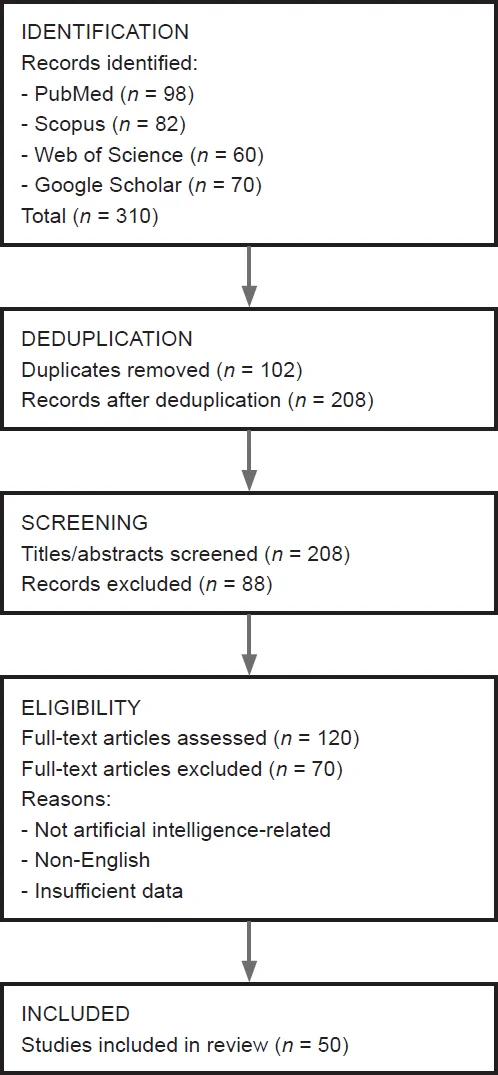
Flow diagram of Preferred Reporting Items for Systematic Reviews and Meta-Analyses.

## Evolving Landscape of Hyperbaric Oxygen Therapy

The evidence base for core indications is strong. For carbon monoxide poisoning, randomized trials show reduced delayed neurologic sequelae after HBOT.1 In chronic radiation injury, controlled trials and systematic reviews indicate improved symptom control and tissue healing.^2^,^3^ Diabetic foot ulcers benefit under specific conditions, with improved healing and fewer amputations in selected patients.^4^,^5^ Beyond these established uses, exploratory and translational studies report neurocognitive gains after mild traumatic brain injury and functional improvements post-stroke, pointing to neurorestorative effects.^6^,^7^ In inflammatory bowel disease, immunomodulation of HBOT has been summarized in a systematic review.^8^ Nevertheless, variability in pressure (1.3–2.5 atmospheres absolute), session duration (60–120 minutes), and total exposures (10–60+) persists across studies and services, undermining comparability and the derivation of unifying guidance.^5^,^9^

Mechanistically, HBOT engages oxidative signaling, angiogenesis, and stem/progenitor cell mobilization; these effects, while biologically plausible, have not yet yielded reliable bedside biomarkers for selecting responders or titrating dose.^6^,^7^,^8^ The gap between mechanistic promise and clinical routine is precisely where AI can help: by learning from multi-modal data to personalize therapy and by enabling real-time adaptation during sessions.

## Lessons from Artificial Intelligence in Medicine

In imaging specialties, deep learning rivals expert performance in tasks such as skin cancer classification and ophthalmic screening.^10^,^11^ In broader clinical medicine, machine learning approaches interpret electronic health records to forecast clinical deterioration and guide therapy, while large, de-identified databases (e.g., Medical Information Mart for Intensive Care-III) have democratized algorithm development.^12^,^13^,^15^ Explainable AI techniques such as SHapley Additive exPlanations can expose the features driving model outputs, providing the transparency clinicians and regulators require.^14^ Digital twins are already piloted in cardiology to simulate hemodynamic responses, and, more generally, in silico trials are being explored to complement prospective evaluations.^15^,^16^

For HBOT, these lessons translate into practical opportunities: train models on multicenter HBOT datasets to suggest personalized pressure–duration–frequency triplets; learn biomarker signatures of response from imaging and omics; detect pre-ictal signatures of oxygen toxicity; and eventually close the loop between sensor streams and chamber controls.^17^

## Pathways to Integrate Hyperbaric Oxygen Therapy and Artificial Intelligence

### Protocol optimization

Historical protocols of HBOT were built on physiological reasoning and clinical tradition. Machine learning can learn from outcome-linked, multicenter HBOT datasets to recommend initial protocols tailored to indication and phenotype. Reinforcement learning — already used to propose sepsis treatment strategies — can adapt regimens across sessions, maximizing benefit while observing safety constraints.^10^,^11^,^12^,^13^ Translational roadmaps emphasize the need for iterative deployment and monitoring to bridge the lab-to-clinic gap.^18^

### Biomarker-driven patient selection

HBOT influences mitochondrial function, angiogenic pathways, and inflammatory set-points. By applying supervised and unsupervised learning to imaging (magnetic resonance imaging/positron emission tomography) and multi-omic panels, it is feasible to identify phenotypes more likely to respond.^6^,^7^,^8^,^19^,^20^ Such models could shift HBOT from trial-and-error to targeted therapy, similar to how precision oncology uses molecular signatures to guide treatment.

### Predictive safety analytics

Central nervous system oxygen toxicity, while uncommon, remains a serious risk. Physiologic monitoring and computational modeling have outlined mechanisms and potential precursors of hyperoxia-induced seizures.^21^,^22^ AI models trained on electroencephalogram, ventilatory parameters, and chamber conditions could provide early warnings, enabling mitigation (e.g., pressure ramp adjustments or session termination). Minimum viable safety stack will include electrodermal activity on volar wrist/thenar; SpO_2_ and chamber logs; ≥ 8–16 Hz electrodermal activity and ≥ 1 Hz vitals; motion/humidity/temperature artifact handling; tonic/phasic electrodermal activity features; target false-alarm rate ≤ 5% per session; two-tier alarms; crew response protocol; site-level monthly audits.

### Adaptive therapy via biosensors

Real-time data streams are the foundation of adaptive control. Advances in wearable and chamber-integrated sensors enable continuous monitoring of photoplethysmography, cerebral oxygenation (near-infrared spectroscopy), respiration, and even biochemical signals in sweat.^23^,^24^,^25^,^26^,^27^ Machine learning inference on these streams can drive decision support, initially advisory dashboards for clinicians, with sufficient validation, closed-loop adjustments of pressure or session duration.

### Digital twins for hyperbaric oxygen therapy

Digital twins, patient-specific computational replicas, can simulate oxygen transport, perfusion, and tissue metabolism under different HBOT regimens. When calibrated with per-session sensor data, predictions of the twin improve over time, supporting pre-treatment planning and adaptive adjustments.^15^,^16^ As in cardiology, such twins can move from descriptive to prescriptive use.

Target indication: high-risk diabetic foot ulcers; Primary endpoint: 12-week wound healing; Update cadence: per session and weekly; Early stop/continue: stop if calibration slope < 0.7 or alarm burden > 8% of sessions; expand if slope 0.9–1.1, Brier ≤ baseline, and safety non-inferior.

### Learning health systems

Robust AI needs big, clean data. Medical Information Mart for Intensive Care-III exemplifies how open critical-care datasets catalyze progress,^28^ and the Observational Health Data Sciences and Informatics community shows how common data models support large-scale observational research.^29^ In HBOT, interoperable registries with harmonized variables and transparent data-quality reporting can sustain model training, external validation, and performance monitoring.^30^,^31^ Implementation science reminds us that workflow fit and user engagement are as critical as algorithmic accuracy.^32^,^33^

## Ethical, Regulatory, and Implementation Considerations

Bias in training data can propagate inequities in access or outcomes. Ensuring representative datasets and auditing model performance across subgroups are minimum requirements for responsible HBOT-AI development.^12^,^20^ Explainable AI should be embedded from the outset; interpretable models or post-hoc explanations enhance trust and ease regulatory review.^14^,^20^ Regulators categorize AI that influences treatment parameters as high-risk Software as a Medical Device, requiring pre-market evidence, change-control plans, cybersecurity, and post-market surveillance.^34^ The evolving European AI Act adds horizontal obligations for transparency, risk management, and human oversight.^35^

Methodological rigor also matters. Preferred Reporting Items for Systematic Reviews and Meta-Analyses 2020 informs transparent synthesis of evidence.^36^ Consolidated Standards of Reporting Trials-AI and Standard Protocol Items: Recommendations for Interventional Trials-AI extensions specify how to design and report clinical trials of AI-enabled interventions.^37^,^38^ while DECIDE-AI provides a preclinical clinical-evaluation framework bridging development to trials.^39^ Continuous performance monitoring in real-world settings helps detect dataset shift and maintain safety.^40^,^41^ Finally, reporting guidelines for prediction models (Transparent Reporting of a multivariable prediction model for Individual Prognosis Or Diagnosis) remain relevant when models are used to stratify HBOT candidates.^42^ Implementation roadmap is displayed in **Figure 2**.

**Figure 2:**
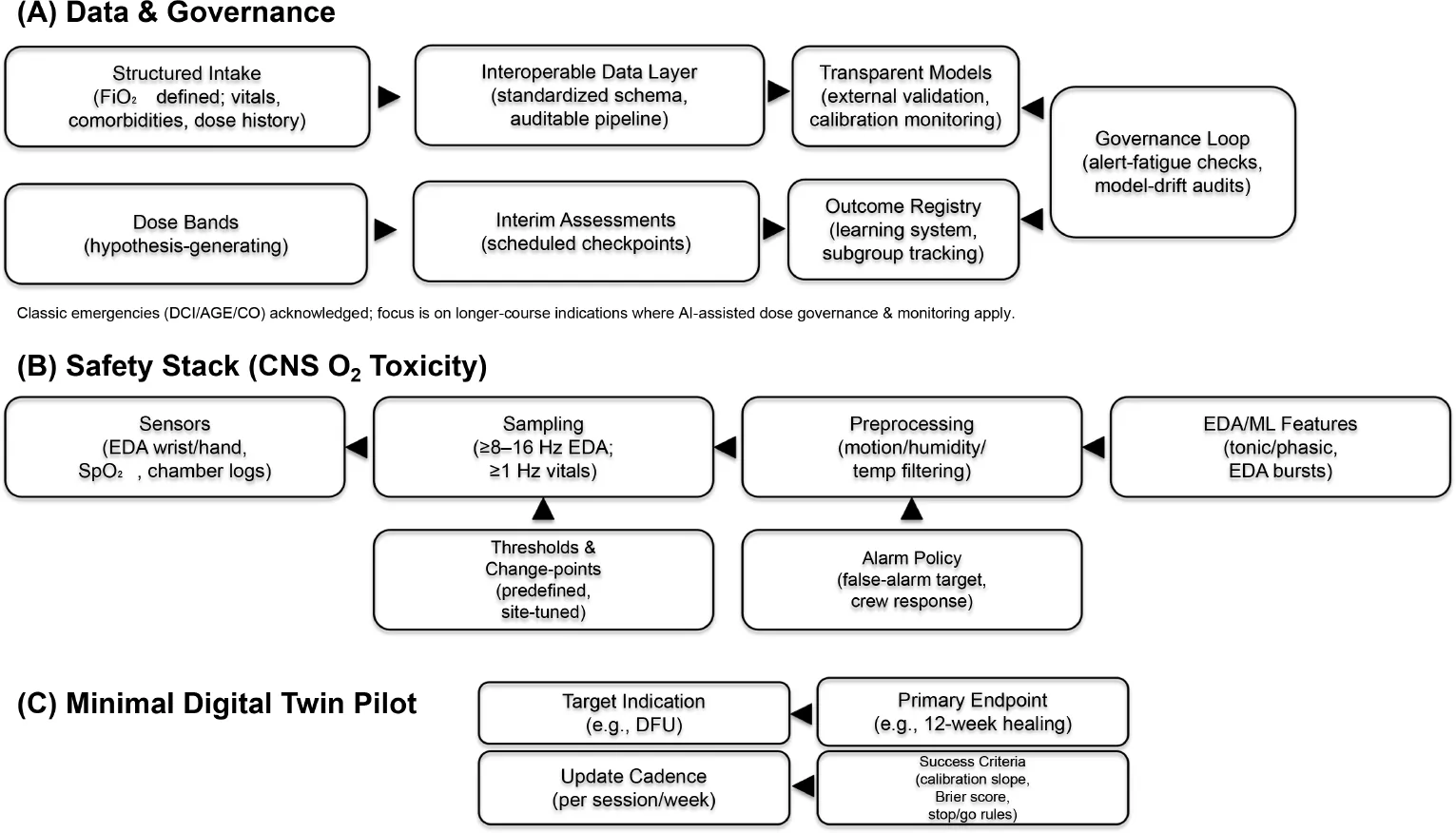
Implementation roadmap for HBOT + AI. (A) Data standardization and governance (intake → interoperable data layer → transparent models → clinician-in-the-loop; dose bands → interim assessments → outcome registry → governance loop). (B) Minimum viable safety stack for CNS oxygen toxicity (sensors → sampling → preprocessing → EDA/ML features → thresholds & alarm policy). (C) Minimal digital twin pilot (target indication, endpoint, update cadence, success criteria). Created with Microsoft PowerPoint. AGE: Arterial gas embolism; AI: artificial intelligence; ATA: atmospheres absolute; CNS: central nervous system; CO: carbon monoxide; DCI: decompression illness; DFU: diabetic foot ulcer; EDA: electrodermal activity; FO_2_: fraction of inspired oxygen; HBOT: hyperbaric oxygen therapy; ML: machine learning; SpO_2_: peripheral oxygen saturation.

## Future Outlook

We envision phased innovation. In the near term, decision-support systems can recommend initial protocols from multicenter data, with clinicians retaining full control. Early pilots should focus on indications with clearer pathophysiology (e.g., late radiation tissue injury) and measurable outcomes (e.g., wound closure trajectories), guided by established HBOT manuals and textbooks.^43^,^44^ Concurrently, mechanistic work on oxidative stress and angiogenesis can inform feature engineering and safety constraints for AI models.^45^

A second phase introduces sensor-informed adaptation: advisory dashboards digest near-infrared spectroscopy, electrocardiography/heart rate variability, and respiratory signals to suggest session-by-session adjustments.^23^,^24^,^25^,^26^ Validation studies should quantify improvements in efficacy, reductions in adverse events, and resource utilization. Legacy literature on chronic wound HBOT and its limitations underscores the need for pragmatic, multi-site designs with standardized endpoints.^46^

Finally, digital twins and federated learning can allow privacy-preserving collaboration across centers, accelerating generalizable models while respecting data governance.^15^,^29^,^47^ Parallel ethical frameworks from World Health Organization and others stress fairness, transparency, and accountability — principles that ought to be embedded throughout the HBOT-AI pipeline.^48^ It is equally important to remain clear-eyed: not all patients will benefit. Observational analyses have reported null effects in some diabetic-foot populations, reminding us to prioritize robust patient selection and prospective validation.^49^ Emerging cellular-level markers (e.g., telomere dynamics and immunosenescence) may complement clinical outcomes in future mechanistic trials.^50^ Observational signals are associations and may reflect confounding by indication; causal interpretations are unwarranted without randomization or rigorous quasi-experimental designs.

## Conclusion

Hyperbaric oxygen therapy has matured from a niche intervention to a versatile modality with clear benefits in several conditions. Yet practice remains constrained by protocol heterogeneity, limited real-time monitoring, and uncertain patient selection. The convergence with AI offers a coherent strategy to address these gaps: learn from multicenter data to optimize protocols; select patients through imaging-omic signatures; safeguard safety with predictive analytics; and progressively adapt therapy using biosensors and digital twins. Advancing toward precision HBOT will require hybrid clinical trials, interoperable registries, explainable and equitable models, and regulatory-grade validation. If pursued thoughtfully, the marriage of oxygen and algorithms can improve outcomes, reduce harms, and illuminate mechanisms of HBOT, transforming a largely empirical therapy into an adaptive, learning intervention. Accordingly, the dose bands proposed in this review are provisional and designed to be falsifiable through rigorous, regulator-aligned evaluation.

## Data Availability

*Not applicable.*
